# Disruption of chromatin organisation causes *MEF2C* gene overexpression in intellectual disability: a case report

**DOI:** 10.1186/s12920-019-0558-8

**Published:** 2019-08-02

**Authors:** Kevin Yauy, Anouck Schneider, Bee Ling Ng, Jean-Baptiste Gaillard, Satish Sati, Christine Coubes, Constance Wells, Magali Tournaire, Thomas Guignard, Pauline Bouret, David Geneviève, Jacques Puechberty, Franck Pellestor, Vincent Gatinois

**Affiliations:** 1 Unité de Génétique Chromosomique, Département de Génétique Médicale, Maladies Rares et Médecine Personnalisée, Hôpital Arnaud de Villeneuve, CHU de Montpellier, Montpellier, France; 2 Cytometry Core Facility, The Wellcome Trust Sanger Institute, Hinxton, Cambridge, UK; 3 Chromatin and Cell Biology Group, CNRS-Institute of Human Genetics, Montpellier, France; 4 Service de Génétique Clinique, Département de Génétique Médicale, Maladies Rares et Médecine Personnalisée, Centre de Référence Anomalies du Développement et Syndromes Malformatifs, Hôpital Arnaud de Villeneuve, CHU de Montpellier, Montpellier, France

**Keywords:** Intellectual disability (ID), Topologically associated domains (TAD), *MEF2C*

## Abstract

**Background:**

Balanced structural variants are mostly described in disease with gene disruption or subtle rearrangement at breakpoints.

**Case presentation:**

Here we report a patient with mild intellectual deficiency who carries a de novo balanced translocation t(3;5). Breakpoints were fully explored by microarray, Array Painting and Sanger sequencing. No gene disruption was found but the chromosome 5 breakpoint was localized 228-kb upstream of the *MEF2C* gene. The predicted Topologically Associated Domains analysis shows that it contains only the *MEF2C* gene and a long non-coding RNA *LINC01226*. RNA studies looking for *MEF2C* gene expression revealed an overexpression of *MEF2C* in the lymphoblastoid cell line of the patient.

**Conclusions:**

Pathogenicity of MEF2C overexpression is still unclear as only four patients with mild intellectual deficiency carrying 5q14.3 microduplications containing *MEF2C* are described in the literature. The microduplications in these individuals also contain other genes expressed in the brain. The patient presented the same phenotype as 5q14.3 microduplication patients. We report the first case of a balanced translocation leading to an overexpression of *MEF2C* similar to a functional duplication.

**Electronic supplementary material:**

The online version of this article (10.1186/s12920-019-0558-8) contains supplementary material, which is available to authorized users.

## Background

Intellectual disability (ID) is a common disorder affecting up to 3% of the population [1]. Between 3 and 15% of patients with ID present numerical or structural chromosomal abnormalities mainly unbalanced rearrangements [2]. Only 0.6% of subjects carry an apparently balanced chromosomal rearrangement such as de novo reciprocal translocations [3].

The link between balanced rearrangements and ID can be explained by several mechanisms such as subtle rearrangement at the breakpoints [2, 4], perturbation of parental imprinting [5], disruption of one or two genes at the breakpoints leading to a loss of function of these genes [6], formation of a fusion gene with a novel function [7] or perturbation of gene expression (previously called positional effect) [8] and, more recently, changes in enhancers or DNA folding modifications within Topologically Associated Domains (TAD) [9, 10].

Separated by specific and robust boundaries, TADs restrict gene expression regulation inside them. Changes in enhancer - promoter interactions and breaking TAD boundaries have been reported to be pathogenic and “TADopathies” constitute an upcoming new category of human mendelian disease [11]. Recent studies showed that disruption in chromatin organization such as TADs can impact gene expression located distantly from breakpoint [12].

In this study, we report the molecular characterization of a de novo balanced reciprocal translocation t(3;5)(p26.3;q14.3) dn in a woman with ID. The breakpoint does not lead to the disruption of a gene but is localised 228-kb upstream of *MEF2C* gene on chromosome 5.

## Case presentation

The proband is the first child of a healthy non-consanguineous couple. Medical family history showed a paternal niece with speech delay, a paternal half-sister with mild ID and a deceased paternal cousin with unspecified malformations.

The girl was born by vaginal delivery after an uneventful pregnancy. Birth parameters were at mean (birth weight: 3.200 kg; birth length: 49 cm; and occipital frontal circumference (OFC) 34 cm). She had global developmental delay diagnosed since 2 years old. She sat at 10 months and learned to walk at 22 months.

At 9 years old, she was diagnosed with attention deficit/hyperactivity disorder and delayed speech. Psychometric evaluation estimated her developmental stage at 3 years for a chronological age of 9 years. She has no autistic or stereotypic features and a unique febrile seizure episode.

Facial features include spread eyebrows, protruding ears with simplified helices and abnormal dermatoglyphics. She also had bilateral fifth finger clinodactyly as her father. Spectroscopic brain MRI, EEG, audition and visual explorations, abdominal ultrasound as well as skeletal X-rays were normal. Urine and blood metabolic screening were also normal.

**Fig. 1:**
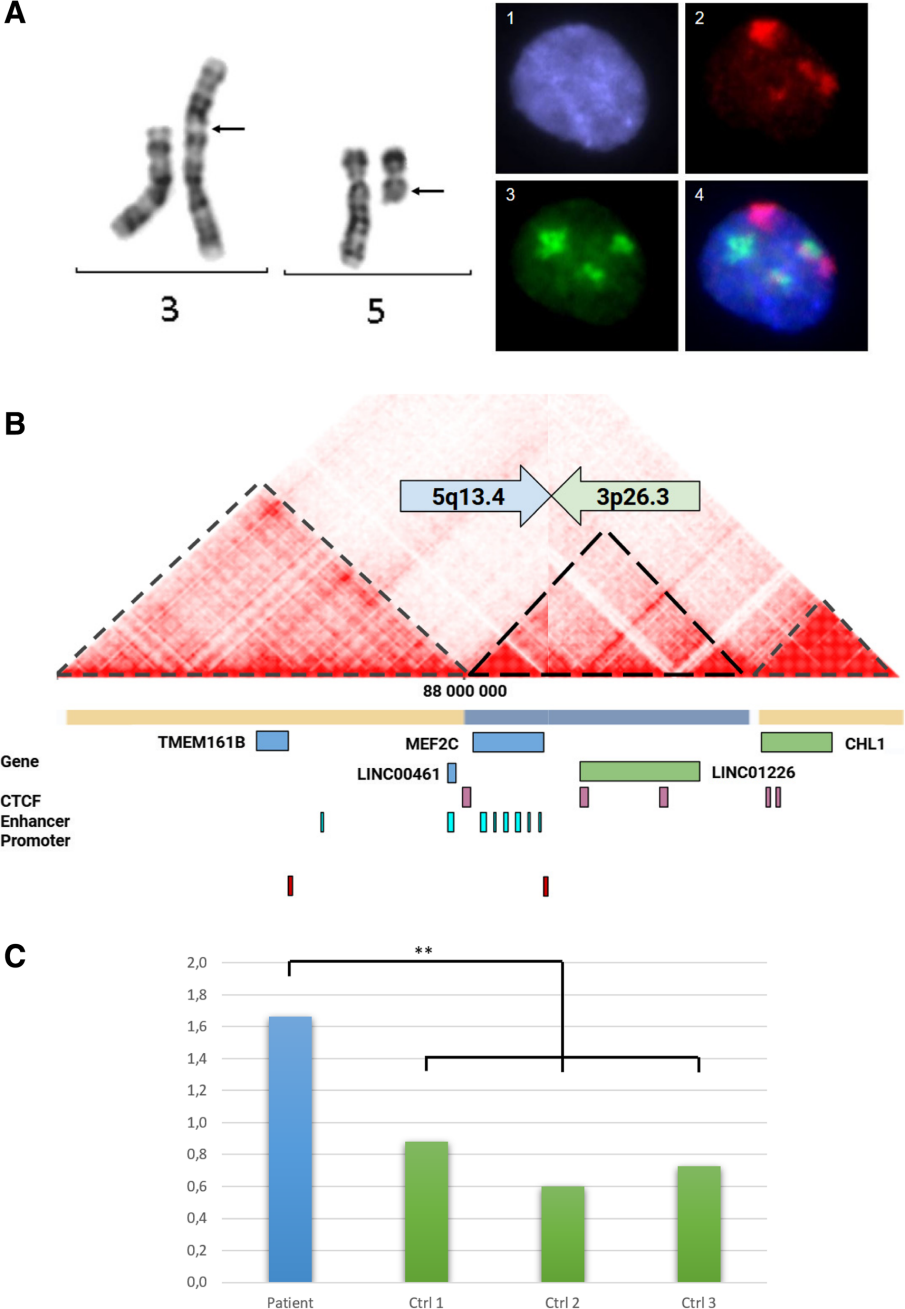
**a** GTG-banding chromosomes 3 and 5 and FISH nucleus assay. Black arrows show chromosome breakpoints on 3p26.3 and 5q14.3. A1. DAPI counterstain (blue). A2. Whole chromosome 3 painting probe (red). A3. Whole chromosome 5 painting probe (green). A4. Merging of A1, A2 and A3. **b** Predicted Hi C maps of der(5) from GM12878 cell line experiment (Liebermann -raw 10 kb) resolution. Black dashed line, yellow and grey bars represent predicted TAD. Blue genes & arrow are in chromosome 5 and green genes & arrow are in chromosome 3. CTCF sites are from ENCODE [13] data. **c** Expression of *MEF2C* gene in the patient’s lymphoblastoid cell lines (blue box) and three normal controls (green boxes), all assay were 3-times repeated, Y-axis shows the MEF2C RNA quantification normalized with the β-2 microglobulin housekeeping gene, ***: *p* < 0.001, One-way ANOVA with *post-hoc* Tukey HSD Test)

The chromosome analysis of the patient and her parents reported a de novo apparently balanced reciprocal translocation 46,XX,t(3;5)(p26.3;q14.3)dn. FISH analysis with chromosome 3 and 5 painting probes showed the unique involvement of chromosomes 3 and 5 in this rearrangement (Fig. 1a).

We confirmed the balanced status of the translocation using a microarray analysis which was normal (100 kb-resolution). Array painting assays and long-range PCR strategy allowed us to perform a fine mapping of these breakpoints. Breakpoints are located at chr3:920,589 and chr5:88,347,198 with the presence of a micro-homology of 3 nucleotides (TGC). No gene was interrupted in these regions. The chromosome 5 breakpoint is localized 228-kb upstream from ATG of the initiator codon of the *MEF2C* gene (NM_001193347). Visualisation of the 3D conformation using the 3D Genome Browser in 7 different cell types allow us to identify reliable TAD boundaries suggesting that the *MEF2C* gene and the *LINC01226* long non coding RNA (lncRNA) exist in the same TAD on chromosome 5 [14]. The TAD on chromosome 3 contains only *CNTN6* and *CETN3* genes (Fig. 1b, Additional file 1: Figure S1 and Additional file 2: Figure S2). RNA studies revealed an overexpression of MEF2C in the patient’s lymphoblastoid cell line compared to 3 controls (gender- and age- matched with the patient) in experiments repeated three times (Fig. 1c). All genomic locations are based on Human Genome Build 37 (hg19).

## Discussion and conclusions

Fine mapping of breakpoints on chromosomes 3 and 5 revealed no gene interruption but a breakpoint on the chromosome 5 localized 228-kb upstream of *MEF2C*.

The *MEF2C* gene causes the syndrome “Mental Retardation, Autosomal Dominant 20” (MIM # 613443) by haploinsufficiency [15]. Balanced translocation in this region have already been described in the literature. Such structural rearrangements on chromosome 5 create a single TAD encompassing *MEF2C,* resulting in decreased MEF2C expression [16]. The pathogenicity of *MEF2C* haploinsufficiency is no longer questioned to explain the phenotype of individuals with severe ID, stereotypic movement and autistic features. However, the pathogenicity of *MEF2C* overexpression is not clearly documented in the literature. Indeed, only 3 children and monochorionic diamniotic twins have been reported with a de novo 5q14.3 microduplication including *MEF2C* [17, 18] and MEF2C overexpression [19]. Interestingly, they share some pathological features such as global development delay with locomotor impairment (Table 1). Other genes included within these microduplications are also expressed in the brain. The major clinical sign described is a mild ID. Pathogenicity of MEF2C overexpression could be partly explained by its interaction on others genes known in human disease. Indeed, *MEF2C* overexpression could lead to *MECP2* and *CDKL5* upregulation [20]*. MECP2* duplication in females is involved in psychiatric symptoms [21] and *CDKL5* duplications are reported in women with heterogeneous symptoms, from learning difficulties to autistic behaviour, developmental delay, language impairment and hyperactivity [22].

**Table 1:** Genomic and clinical features of patients with *MEF2C* duplications compared to the present case

	Genetic features				Clinical features										
	Size (Mb)	Start	End	Inheritance	Ultrasound findings	Neonatal feeding	Microcephaly	Global developmental delay	Locomotor delay	Speech delay	Autistic trait	Eye	Facial features	MRI	Other
Present case	No CNV (balanced translocation)			de novo	–	–	–	+	+	+	–	–	spread eyebrows protruding ears with a fairly simple helix	–	
Le Muer et al. (2010) [18]	4.5	86,142,512	90,712,814	de novo	NA	NA	+	+	+	+	–	NA		–	
Novara F. et al. (2013) [17], patient 1	5.5	85,598,295	91,182,469	de novo	–	poor sucking	+	+	+	+	–	hypermetropia	eye asymmetry metopic prominence occipital asymmetry	asymetric enlargement of lateral ventricles	
Novara F. et al. (2013) [17], patient 2	5.2	87,356,360	92,591,506	de novo	IUGR	poor sucking	+	+	+	–	–	–	wide and flat nasal root smooth filtrum microretrognathia clinodactyly of the 4th and 1st toes	–	persistant aseptic fever
Cesaretti C. et al. (2016) [19]	4.6	86,129,664	90,762,803	de novo	Tw1: mild ventriculomegaly, short CC Tw2: heart bi-ventricular hypertrophy, short CC	NA	NA	NA	NA	NA	NA	NA	NA	NA	

+: present; −: absent; CC: corpus callosum; CNV: copy number variation; IUGR: intrauterine growth retardation; Mb: mega base; MRI: magnetic resonance imaging; NA: not available or not relevant; Tw: twin

In this article, we report the study of a patient who has ID associated with speech delay. According to the breakpoints of the translocation t(3;5), the predicted TAD in chromosome 3 only contains *CNTN6* and *CETN3* gene. Few studies described patients with ID carrying microdeletions/microduplications containing *CNTN6* [23]. Still these CNVs have also been reported in some phenotypically normal individuals in the databases of genomic variants. They are mostly inherited from healthy parents and no patient has been identified with a point mutation of CNTN6 (ClinGen Dosage Sensitivity Map Curation https://www.ncbi.nlm.nih.gov/projects/dbvar/clingen/). To date *CETN3* is not described in human disease. In chromosome 5, we identify a possible new TAD encompassing *MEF2C* and *LINC01266*. Our results of RNA quantification showed a clear significant overexpression of MEF2C in the patient’s lymphoblastoid cell line. Further FISH studies could be performed to completely confirm that *MEF2C* and *LINC01266* are in the same TAD. LncRNAs are known to be involved in cis transcriptional regulation and chromosomal architecture [24]. According to GTEx, *LINC01266* is also expressed in brain tissue [25]*.* No other major regulatory elements such as enhancers are predicted to be in this new TAD [26] . Localisation of the breakpoint is close to those of published cases, thus could not explain the upregulation (Additional file 1: Figure S1). As previously reported cases with balanced translocation around *MEF2C* all lead to a downregulation of the gene [16], our hypothesis is that *LINC01266* could be involved in the upregulation of *MEF2C*.

To summarize, we report a disruption of chromatin organisation caused by balanced translocation t(3;5) with chromosome 5 breakpoint upstream of the overexpressed *MEF2C* gene, probably responsible for the patient’s phenotype. This case report adds substantial evidence of a specific phenotype associated with the overexpression of *MEF2C*.

## Additional file

Additional file 1: **Figure S1.** Localisation of breakpoints on Hi C maps from GM12878 cell line experiment on chromosome 3 and chromosome 5 (Liebermann -raw 10 kb resolution). Grey arrow represents the breakpoint localisation. Dashed blue arrow represent other breakpoint described by Redin et al. (Redin et al. [16]) with MEF2C downregulation. Blue genes & arrow are in chromosome 5 and green genes & arrow are in chromosome 3. (PNG 355 kb)

Additional file 2: **Figure S2.** Chromosome 5 TAD boundaries across 4 different cell types (IMR90, NHEK, GM12878 and KBM7). Black dashed line, yellow and grey bars represent TADs. Grey arrow represents the breakpoint localisation. Black line represents TAD boundary. (PNG 257 kb)

Additional file 3 Materials and methods. (DOCX 15 kb)

## Data Availability

The datasets used and/or analysed during the current study are available from the corresponding author on reasonable request. The main method descriptions is available in the Additional file 3.

## Abbreviations

der: derivative chromosome
EEG: ElectroEncephaloGraphy
ID: Intellectual Disability
kb: kilobase
Lnc: Long non coding
MRI: Magnetic Resonance Imaging
OFC: Occipital Frontal Circumference
t: Translocation
TAD: Topologically Associated Domains
