# Supplementary figures and images for: Disruption of chromatin organisation causes MEF2C gene overexpression in intellectual disability: a case report
Source: BMC Med Genomics. 2019 Aug 2;12:116. doi: 10.1186/s12920-019-0558-8 (PMC6679470; doi:10.1186/s12920-019-0558-8)

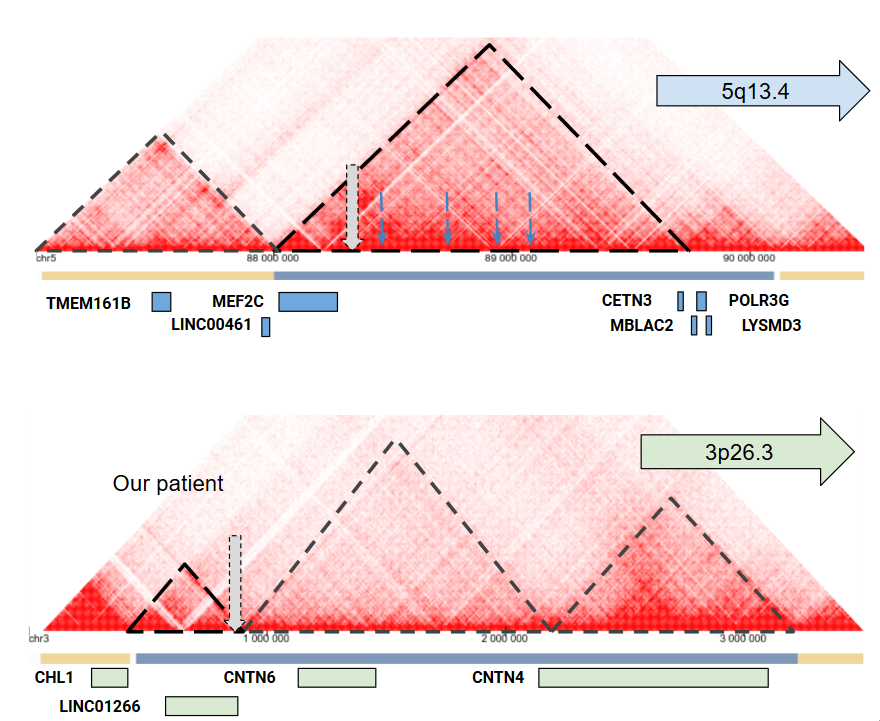

Supplement: Supplementary file 1 — Figure S1. Localisation of breakpoints on Hi C maps from GM12878 cell line experiment on chromosome 3 and chromosome 5 (Liebermann -raw 10 kb resolution). Grey arrow represents the breakpoint localisation. Dashed blue arrow represent other breakpoint described by Redin et al. (Redin et al. [16]) with MEF2C downregulation. Blue genes & arrow are in chromosome 5 and green genes & arrow are in chromosome 3. (PNG 355 kb) [file 12920_2019_558_MOESM1_ESM.png]

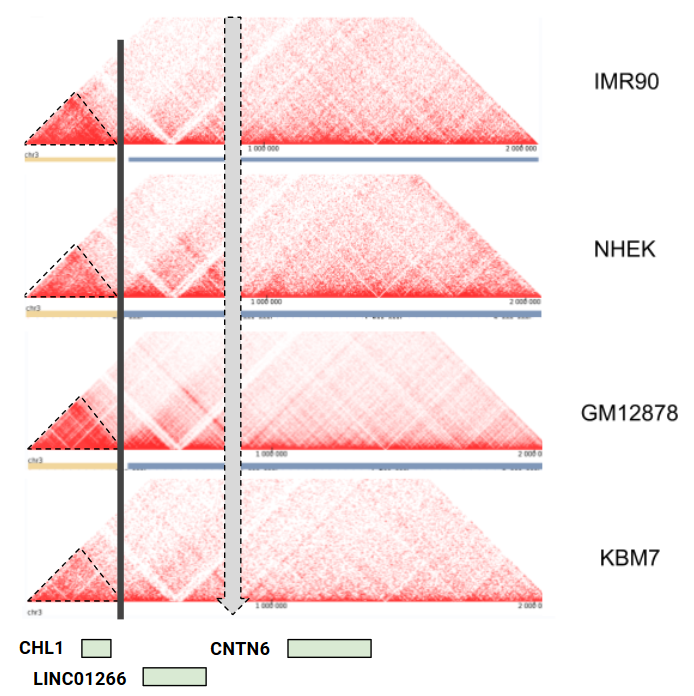

Supplement: Supplementary file 2 — Figure S2. Chromosome 5 TAD boundaries across 4 different cell types (IMR90, NHEK, GM12878 and KBM7). Black dashed line, yellow and grey bars represent TADs. Grey arrow represents the breakpoint localisation. Black line represents TAD boundary. (PNG 257 kb) [file 12920_2019_558_MOESM2_ESM.png]
