# Supplementary material for: Disruption of chromatin organisation causes MEF2C gene overexpression in intellectual disability: a case report
Source: BMC Med Genomics. 2019 Aug 2;12:116. doi: 10.1186/s12920-019-0558-8 (PMC6679470; doi:10.1186/s12920-019-0558-8)
Supplement: Supplementary file 3 — Materials and methods. (DOCX 15 kb) [file 12920_2019_558_MOESM3_ESM.docx]

**Additional file 3**

**Materials and methods**

*Cytogenetics*

Conventional and molecular cytogenetic analyses were performed from culture of lymphocytes according to standard techniques. Specific chromosomal painting probes (Poseidon, Amsterdam, The Netherlands) were used for additional FISH techniques.

*Microarray analysis*

High-resolution microarray analysis was realized with GeneChip® Human Mapping 6.0 SNP Array kit (Affymetrix, High Wycombe, UK). Data were processed by GTC 4.2 software (GenoTyping Console, Affymetrix). Homemade and international databases were used for Copy Number Variations (CNVs) annotations.

*Array painting (AP)*

The derivative chromosomes were isolated as described previously (Gribble SM et al., Nat Protoc. 2009;4:1722–36.) using a flow sorter (MoFlo®, Beckman Coulter). After extraction, derivative chromosome DNA was hybridized on the high resolution GeneChip® Human Mapping Cytoscan HD Array (Affymetrix). Extraction and interpretation of data were performed with the ChAS 2.1 software (Affymetrix). The chromosome breakpoint corresponded to a transition between a high and a low log_2_ ratio.

*Sanger sequencing*

Sanger sequencing to precise the mapping of breakpoints pinpointed by AP was performed after a long range polymerase chain reaction (PCR) (GoTaq® long PCR Master kit, Promega, France). Primers were designed according to the observed transition during AP analysis (data not shown). Sanger reaction was performed with usual clinical laboratory procedures and with BigDye™ Terminator v3.1 (Applied Biosystems). The sequences were obtained by running on Hitachi 3130x/Genetic Analyser (Applied Biosystem). The sequences spanning the junctions are aligned on hg19 reference, using BLAST (Kent WJ. Genome Res. 2002;12:656–64).

*3D Genome and TAD analysis*

We use the 3D Genome Browser coupled with the UCSC Genome Browser to analyse TAD with Hi-C maps from differents cell types in order to select only evolutionary conserved TAD boundaries. All details about our pipeline based on public online tools is published (Yauy K. et al.Methods in Molecular Biology. 2018. p. 353–61).

*Reverse transcription-polymerase chain reaction (RT-qPCR)*

The expression of MEF2C gene was quantified in lymphoblastoid cell line by RT-qPCR according to standard procedures using primers Mef2C-F (5'-CGAGATGCCAGTCTCCATCC-3') and Mef2C-R (5'-AGCAGACCTGGTGAGTTTCG-3'), located in exons 7 and 9, respectively. Quantification was performed with a LightCycler 480 (Roche®). The statistical test used for interpretation was a Welch Two Sample t-test with three controls in triplicate. Experiments were realized three times.
